# Putative SARS-CoV-2 M^pro^ Inhibitors from an In-House Library of Natural and Nature-Inspired Products: A Virtual Screening and Molecular Docking Study

**DOI:** 10.3390/molecules25163745

**Published:** 2020-08-17

**Authors:** Stefania Mazzini, Loana Musso, Sabrina Dallavalle, Roberto Artali

**Affiliations:** 1 Department of Food, Environmental and Nutritional Sciences, Università degli Studi di Milano, via Celoria 2, I-20133 Milano, Italy; stefania.mazzini@unimi.it (S.M.); loana.musso@unimi.it (L.M.); 2 Scientia Advice di Roberto Artali, 20832 Desio, MB, Italy; roberto.artali@scientia-advice.com

**Keywords:** COVID-19, coronavirus, infectious diseases, natural products library, molecular docking, molecular modeling

## Abstract

A novel coronavirus (severe acute respiratory syndrome coronavirus 2, SARS-CoV-2) has been the cause of a recent global pandemic. The highly contagious nature of this life-threatening virus makes it imperative to find therapies to counteract its diffusion. The main protease (M^pro^) of SARS-CoV-2 is a promising drug target due to its indispensable role in viral replication inside the host. Using a combined two-steps approach of virtual screening and molecular docking techniques, we have screened an in-house collection of small molecules, mainly composed of natural and nature-inspired compounds. The molecules were selected with high structural diversity to cover a wide range of chemical space into the enzyme pockets. Virtual screening experiments were performed using the blind docking mode of the AutoDock Vina software. Virtual screening allowed the selection of structurally heterogeneous compounds capable of interacting effectively with the enzymatic site of SARS-CoV-2 M^pro^. The compounds showing the best interaction with the protein were re-scored by molecular docking as implemented in AutoDock, while the stability of the complexes was tested by molecular dynamics. The most promising candidates revealed a good ability to fit into the protein binding pocket and to reach the catalytic dyad. There is a high probability that at least one of the selected scaffolds could be promising for further research

## 1. Introduction

The new SARS-like severe pneumonia illness was first identified among the workers in the Huanan Fish and Seafood market in Wuhan, Hubei province, in December 2019 [1]. Sequencing of the complete genome on 13 January 2020 showed that it was a novel coronavirus (GenBank No. MN908947) [2], initially designated by the preliminary name 2019-nCoV and then with the official name of SARS-CoV-2 (severe acute respiratory syndrome coronavirus 2), while the disease caused by SARS-CoV-2 has been termed Coronavirus disease 2019 (COVID-19), and later evolved into a global pandemic [3].

**Figure 1:**
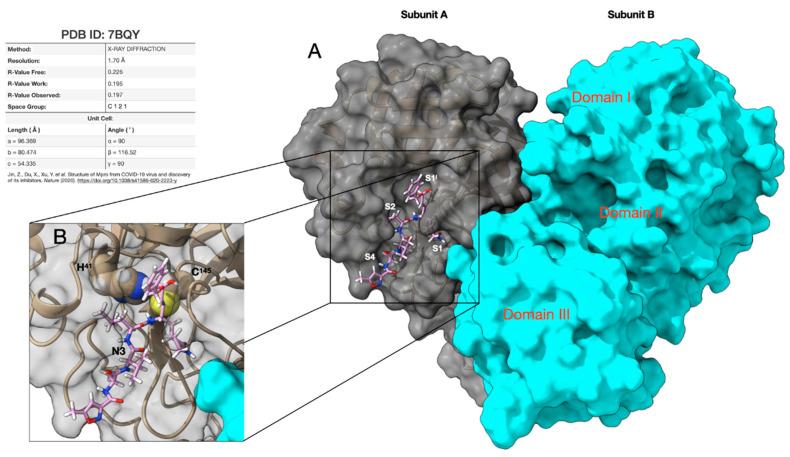
Structure of the SARS-CoV-2 M^pro^ used in this work (subunit A) as a target for virtual screening and molecular docking. (**A**) 3D representation of the SARS-CoV-2 main protease dimer complexed with the inhibitor N3 showing the three domains (I–III) and the binding pockets S1^I^, S1, S2, and S4. Subunits A (silver) and B (light blue) are drawn as solvent accessible surfaces (SAS) while N3 is rendered in a stick. (**B**) Particular of the catalytic dyad composed of the C^145^ and H^41^ residues rendered in CPK (Corey-Pauling-Koltun). The thiol group of C^145^, belonging to the S1^I^ pocket, anchors the N3 inhibitor by a covalent linkage, thus allowing it to maintain the activity. Protein data bank (PDB) accession code, data collection, sand refinement statistics of the structure are summarized in Table S1.

The SARS-CoV-2 virus belongs to the family of *Coronaviridae*, and spreads widely among humans and other mammals, causing a wide range of infections from common cold symptoms to fatal diseases, such as severe respiratory syndrome [4]. SARS-CoV-2 is an enveloped positive-sense single-stranded RNA (ssRNA) virus, consisting of 29,903 nucleotides and two untranslated sequences of 254 nucleotides at the 5′ and 229 nucleotides at the 3′ ends, which compare it to SARS-CoV and other coronaviruses [5,6,7]. SARS-CoV-2 enters human cells via binding of the viral spike protein to the human angiotensin-converting enzyme 2 (ACE2). The replicase genes encode for two polyproteins required for correct viral replication and transcription [8]. Through an extensive proteolytic process, the functional polypeptides are released from these two polyproteins (pp1a and pp1ab). The proteolytic process is mainly conducted by a papain-pike protease (PL^pro^), which performs three cleavages, and by a 33.8 kDa main protease (M^pro^), also called 3C-like protease, which is responsible for the remaining 11 cuts, leading to the formation of non-structural proteins (NSPs). Thus, as the recognition sequence of M^pro^ is not recognized by any host protease, this enzyme represents an optimal target for the search of inhibitors potentially usable as drugs in the treatment of SARS-CoV-2 infections. Thanks to the efforts of several structural biology and crystallography research groups, we currently have access to a significant number of SARS-CoV-2-related protein structures (like spike protein, papain-like, and main protease) in the Protein Data Bank (see Table S1, Supplementary Materials) [9]. In this work, we use the crystal structure of SARS-CoV-2 M^pro^ at 1.7 Å resolution (PDB id. 7BQY) [10], with the asymmetric unit containing only one polypeptide in the complex with the inhibitor N3. Two of these polypeptides (designated as subunit A and B) associate to form a dimer by a crystallographic two-fold symmetry axis. Each subunit is composed of three domains. Domains I (residues 8–101) and II (residues 102–184) have an antiparallel β-barrel structure. Domain III (residues 201–303) contains a globular cluster of five helices and is connected to domain II by means of a long loop region (residues 185–200). It is involved in regulating the dimerization of the M^pro^, mainly through a salt-bridge interaction between E^290^ of one protomer and R^4^ of the other. The tight dimer formed by SARS-CoV-2 M^pro^ has a wide contact interface, predominantly between domain II of the subunit A and the NH_2_-terminal residues (“N-finger”) of the subunit B, with the two molecules oriented perpendicular to one another (Figure 1A).

The active sites of M^pro^ are highly conserved among all CoVs and are usually composed of four pockets (S1^I^, S1, S2, and S4), arranged as shown in Figure 1A [11]. In this respect, dimerization represents a necessary step for catalytic activity. Indeed, the N-finger of each of the two subunits interacts with E^166^ of the other subunit, and thereby helps shape the S1 pocket of the binding site, which also involves S1^I^ from subunit B. At the active site of SARS-CoV-2 M^pro^, C^145^ and H^41^ (Cys-His) form a catalytic dyad, where the thiol of C^145^, belonging to the S1^I^ pocket, anchors the inhibitors by a covalent linkage, thus allowing them to exert their antiviral activity (Figure 1B). The S2 pocket of M^pro^ is large enough to accommodate bulky fragments (like ring systems), while the presence of functional groups capable of forming hydrogen bonds with the residues of the S4 pocket could be useful to improve the interaction with the protease.

To date, only Remdesivir has been fully authorized by EMA (European Medicines Agency) to treat SARS-CoV-2, while no vaccines have yet been authorized by regulatory authorities. Across the world, several clinical studies are ongoing to test the safety and efficacy of treatments and vaccines [12,13]. Alternative approaches from traditional Chinese medicine have been reported as well [14,15]. In any case, the current clinical practice to manage this pandemic emergency is based on symptom-based therapies [16,17]. Therefore, the scientific community is accelerating the discovery of suitable drugs that can be used to treat COVID-19 patients. In this context, although the process of drug discovery is long and complicate, computational chemistry techniques can give precious help. One of these techniques is certainly virtual screening, which can be used to screen both new molecules and approved drugs. Regarding approved drugs, they can represent an important starting point to reduce the time of the drug discovery process through repurposing, as these drugs have already been tested for efficacy/safety, and the label indication could be extended. Another invaluable source of “leads to drugs” are natural products (antibiotics, marine compounds, phytochemicals), which are considered “privileged scaffolds” in the search for new drug candidates [18]. Having evolved over millennia to acquire specific ligand-protein binding motifs, these compounds are structurally predisposed to interact with biomolecules. In addition, natural compounds possess enormous chemical diversity, unsurpassed by any available synthetic screening libraries. Considering that three-quarters of the drugs for all diseases worldwide over the past half-century derived from natural molecules [18], it is clear the key role of natural compounds as a source of inspiration in drug discovery.

In recent months, many research groups have made use of virtual screening and molecular docking techniques for the repurposing of libraries containing approved compounds [19,20,21,22], natural compounds [23,24,25,26], on-line databases [27,28,29,30], and in-house compound libraries [31].

The aim of the present study was to identify anti-SARS-CoV-2 candidates using a combined approach of virtual screening and molecular docking techniques. The information deriving from the screening will be useful in the investigation of new, potentially effective and selective inhibitors of the SARS-CoV-2 M^pro^ target.

## 2. Results

**Scheme 1:**
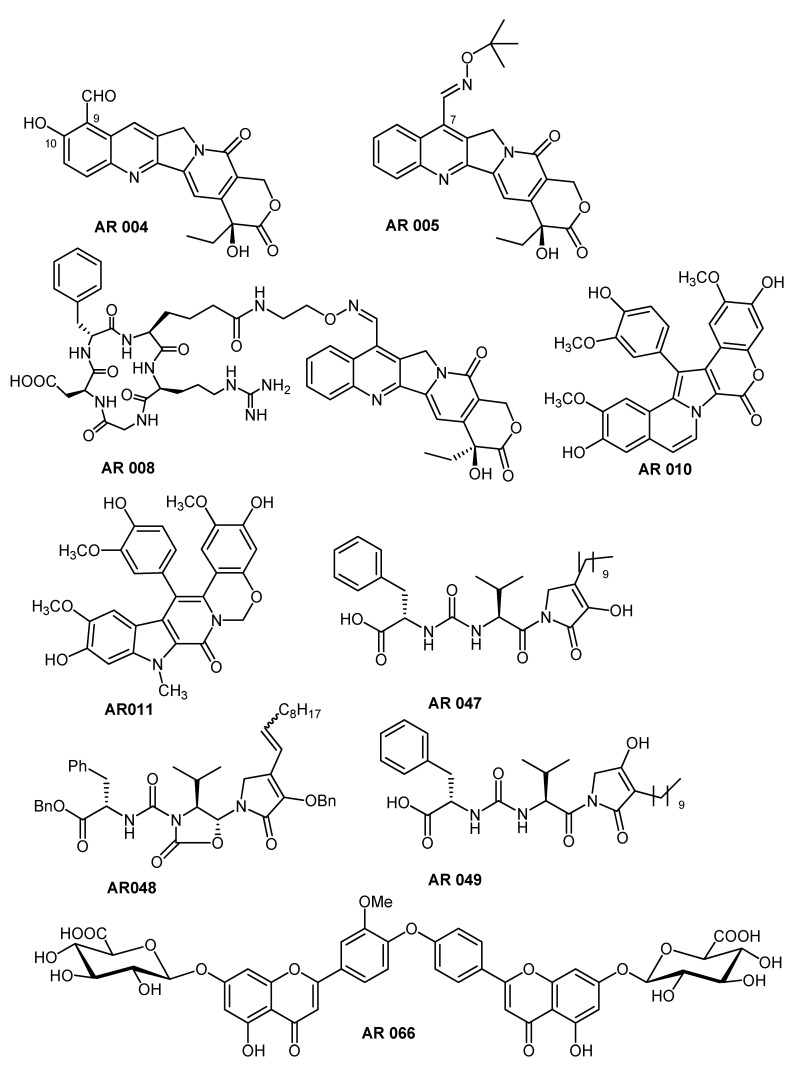
Structures of the selected compounds.

We initially used virtual screening to test our in-house library of natural compounds and synthetic analogs (isolated or prepared by our research group) on the SARS-CoV-2 M^pro^ target. This library consists of more than one hundred structurally diverse small molecules having various core scaffolds and different patterns of substitution, to cover a wide range of biologically relevant chemical space. In addition to significant chemical diversity, the screened compounds also have quite different sizes, with molecular weights in the range 190–1035, in order to investigate the ability to fit into the protein binding pocket and to reach the catalytic dyad. Relevant information about the compounds in our in-house library are reported in Tables S2 and S3 (Supplementary Materials). Using the criteria outlined above, virtual screening allowed us to select a small group of potentially active molecules on SARS-CoV-2 M^pro^. After careful analysis of the interactions of these molecules with the protease enzymatic site, we decided to focus our attention on four classes of compounds (Scheme 1). Their conformations were then re-scored with AutoDock obtaining the final poses, which will be discussed below.

**Figure 2:**
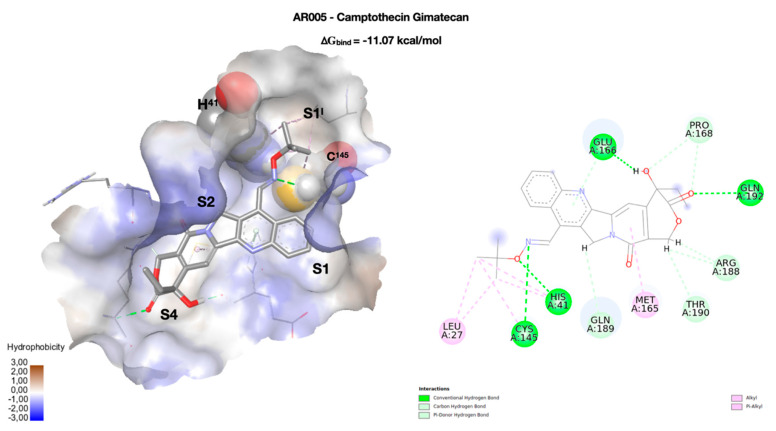
Docking pose of the camptothecin derivative gimatecan (AR005). On the left, the conformation of the ligand (in stick) inside the enzymatic binding site, represented as solvent accessible surface (SAS) and colored according to the hydrophobicity of the residues (in wireframe). The two key residues of the catalytic dyad (H^41^ and C^145^) are rendered in CPK. The four binding pockets (S1^I^, S1, S2, and S4) occupied by the molecule are highlighted. On the right, a two-dimensional representation of the molecular interactions between the ligand and the enzyme. Here, the colors used refer to the different interactions, as indicated in the legend.

The first class of compounds taken into consideration includes a small collection of analogs of the natural compound camptothecin (CPT), a potent inhibitor of the enzyme topoisomerase I. Most of the studied analogs in our laboratory have a group linked to position 7 of the CPT scaffold, which is a 7-oxyiminomethyl moiety [32], a polyamino chain [33], a diaminedichloro-platinum (II) complex [34], or an arginine–glycine–aspartic acid (RGD) motif [35]. Due to its size and structure, the CPT-RGD conjugate (AR008) shows the best interaction with the enzyme binding site, but precisely because of its chemical-physical characteristics (see Table S3) it can be hardly considered either a drug or a lead-like compound. In this respect, the two analogs of the camptothecin AR004 and AR005 (gimatecan) are more promising, as both compounds can be considered both drug- and lead-like. Focusing on AR005, the tetracyclic nucleus of the CPT interacts with the residues of the S1 and S4 binding pockets, mainly through two hydrogen bonds with Q^192^ (3.05 Å) and E^166^ (2.42 Å), which also interacts with the aromatic nitrogen of CPT (Figure 2). This orientation of the tetracyclic system is common to all studied CPT derivatives, contributing strongly to the geometry of the complex. Hydroxylamine nitrogen forms a hydrogen bond with C^145^ (3.25 Å), one of the two residues that make up the catalytic dyad, while the other catalytic residue (H^41^) forms a hydrogen bond with hydroxylamine oxygen, at a distance of 2.89 Å. The S2 binding pocket is only partially occupied by the carbonyl group of the polycyclic system. The network of interactions is completed by weaker ones, which, together with the interactions with the polycyclic system, and above all with the catalytic dyad, make CPT a promising scaffold for the construction of new SARS-CoV-2 protease inhibitors. Although none of the derivatives approach the ∆G_bind_ of CPT-RGD (−15.09 kcal/mol), their chemical-physical characteristics make them perfect starting points for further studies.

**Figure 3:**
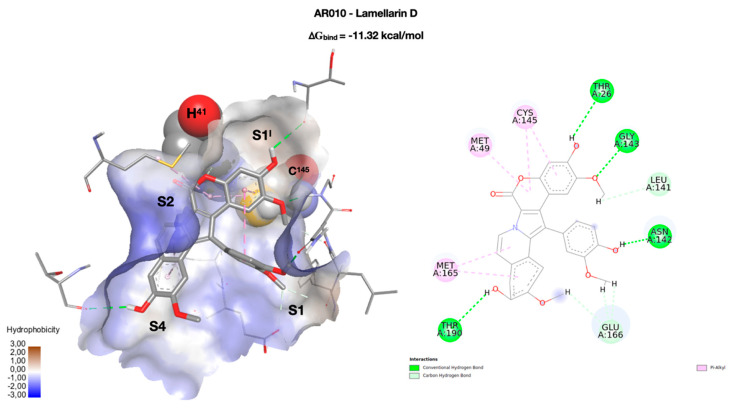
On the left, the best docking conformation of lamellarin D (AR010 in stick) inside the enzymatic binding site. The binding site is represented as a solvent accessible surface (SAS) colored according to the hydrophobicity of the residues (wireframe). The two key residues of the catalytic dyad (H^41^ and C^145^) are rendered in CPK, and the four binding pockets (S1^I^, S1, S2, and S4) occupied by the molecule are highlighted. On the right, a two-dimensional representation of the molecular interactions between the ligand and the enzyme. The colors used refer to the different interactions, as indicated in the legend.

The natural compound lamellarin D and its analog (AR010 and AR011, respectively, Scheme 1) are endowed with a wide variety of biological activities, including antitumor, antibiotic, and Human Immunodeficiency Virus (HIV-1) integrase inhibitory activity [36]. The pseudo-planar system of lamellarin D (AR010) is arranged as in Figure 3, with the three methoxy-substituted rings positioned in the binding pockets S1^I^, S1, and S4. The system is held in place by four h-bonds which, moving from S1^I^ to S4, involve the residues T^26^ (3.35 Å), G^143^ (2.84 Å), N^142^ (2.48 Å), and T^190^ (3.35 Å). The residue of the catalytic dyad C^145^ interacts with the rings of the coumarin portion through a pi-alkyl interaction, while no interactions with H^41^ have been observed. S2 is occupied by the central portion of the pentacyclic system. Lamellarin D has a very promising score (∆G_bind_ = −11.32 kcal/mol) and passes the Rule of 5 (RO5) but not the Oprea’s lead-like test.

**Figure 4:**
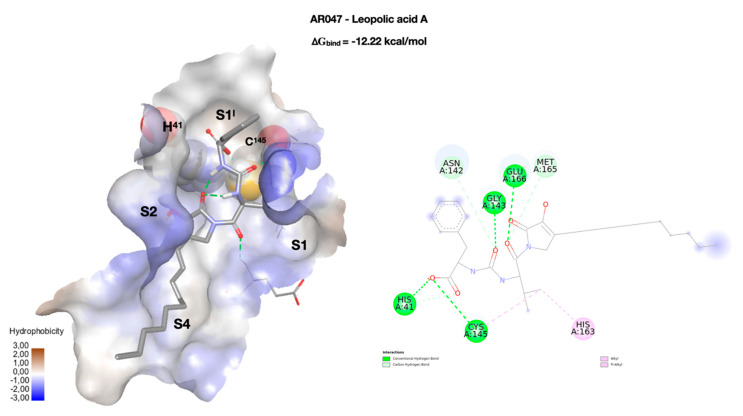
Docking pose of leopolic acid A (AR047) inside the enzymatic binding site, were the two key residues of the catalytic dyad (H^41^ and C^145^) are rendered in CPK. The compound is rendered in stick, while the binding site is represented as the solvent accessible surface (SAS), colored according to the hydrophobicity of the residues (wireframe). The four binding pockets (S1^I^, S1, S2, and S4) occupied by the molecule are highlighted. On the right, a two-dimensional representation of the molecular interactions between the ligand and the enzyme. The colors used refer to the different interactions, as indicated in the legend.

Leopolic acid A (AR047, ∆G_bind_ = −12.22 kcal/mol), a natural compound obtained from a terrestrial-derived *Streptomyces* sp. isolated from the rhizosphere of the plant *Juniperus excels*, was synthesized for the first time by our group in 2016. The compound possesses a rare 2,3-pyrrolidinedione nucleus linked to an ureido dipeptide [37]. Together with its analogs (AR048 and AR049), leopolic acid A has a different pattern of interactions compared to the previous compounds (Figure 4). The ring of the phenylalanine portion is positioned in S1^I^, and its carboxyl group forms two h-bonds with the residues of the catalytic dyad, H^41^ (2.66 Å) and C^145^ (2.88 Å). The ureic keto group forms an h-bond with G^143^ at 2.76 Å, while the isopropyl group points towards pocket S1. The complex is stabilized by an additional h-bond with G^166^ (3.20 Å). The S2 and S4 pockets are occupied by the 2,3-pyrrolidinedione ring, and by the aliphatic lateral chain, respectively. This arrangement is common to all derivatives of this class, with the only exception of AR048 where the side chain, which has a double link, folds back, pointing towards S1. Both derivatives could be considered drug-like, but not lead-like. This behavior is mainly due to the presence of the aliphatic side chain, and therefore, to the high number of rotatable bonds. Replacing this moiety with a more rigid system could certainly improve their characteristics as a lead.

**Figure 5:**
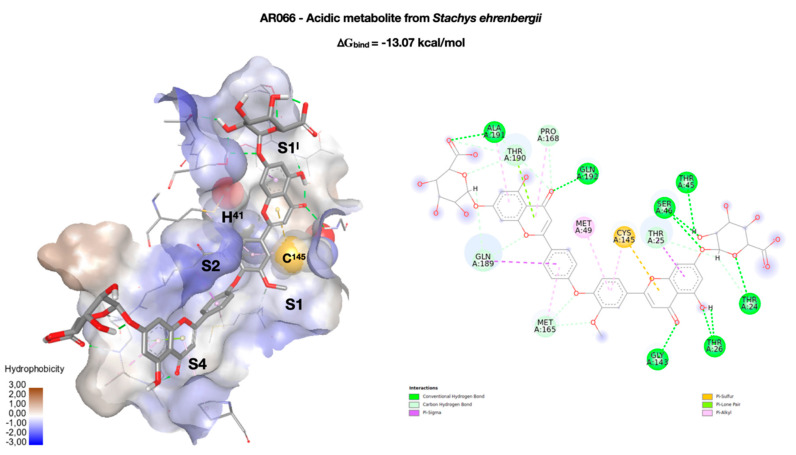
Best docking pose of an acidic metabolite isolated from the aerial parts of *Stachys ehrenbergii* (AR066). On the left, the conformation of AR066 (in stick) inside the enzymatic binding site, represented as the solvent accessible surface (SAS), and colored according to the hydrophobicity of the residues, rendered in wireframe. The two key residues of the catalytic dyad (H^41^ and C^145^) are rendered in CPK. The four binding pockets (S1^I^, S1, S2, and S4) occupied by the molecule are highlighted. On the right, a two-dimensional representation of the molecular interactions between the ligand and the enzyme. The colors used refer to the different interactions, as indicated in the legend.

Last but not least, we had selected the natural compound AR066, a novel acidic metabolite isolated from the aerial parts of *Stachys ehrenbergii* in 2011 [38]. Unlike the previous compound, in this case the molecule stretches completely along the groove that goes from S1^I^ to S4 (Figure 5). Starting from S1^I^, the glycoside group forms four hydrogen bonds with T^24^ (3.47 Å), T^45^ (2.91 Å), and S^46^ (2.87 and 2.99 Å). The phenolic OH of the benzopyran ring forms an h-bond with T^26^ (1.97 and 2.03 Å), while the carbonyl group forms a hydrogen bond with G^143^ (3.69 Å). The same ring is also involved in a sulfur–π interaction with the C^145^ of the catalytic dyad. The methoxy group of the other aromatic ring partially occupies the pocket S2, while the group C=O of the second benzopyrane ring forms an h-bond with Q^192^ (2.88 Å). Finally, the second glycoside group anchors the molecule to the S4 side by means of an h-bond with A^191^ (2.69 Å). As in the case of CPT-RDG, the complex network of interactions contributes to the overall stability of the complex (∆G_bind_ = −13.07 kcal/mol) but limits its use both as a drug and as a lead. The compound is in any case very interesting, as it is placed in the binding site like many of the ligands present in the PDB deposited structures. The analysis of the complex could, therefore, provide useful indications for the design of specific inhibitors for SARS-CoV-2 M^pro^.

The stability of the four complexes with SARS-CoV-2 M^pro^ was studied by molecular dynamics (MD). The results show that all the complexes, with the exception of the one with AR066, keep quite unchanged all the previously discussed interactions, showing only slight fluctuations in the interaction distances. In the case of the complex between AR066 and SARS-CoV-2 M^pro^, the hydrogen bond with A^191^ is lost, and is replaced by a hydrogen bond with a water molecule. In this regard, the solvent molecules interact with the complexes forming transient and unstable hydrogen bonds, which, however, do not lead to noteworthy conformational variations. In addition, no solvent molecules have shown the ability to form bridges between the ligands and the enzyme. The stability of the complexes is also confirmed by the average values of the Root-Mean-Square Deviations (RMSD) and the Root-Mean-Square Fluctuations (RMSF) calculated for the four complexes during the simulation time. The deviations that occurred during the MD simulation describe the stability of the conformations, and the small deviations of the RMSD mean values observed in the four complexes reflect their stable nature. Similarly, the RMSF values describe the conformational changes of the enzyme due to binding with ligands. Again, the small average RMSF values obtained during the simulation show that the secondary structure of the enzyme remains stable during the simulation. The average values of RMSD and RMSF obtained in the four 1.0 ns MD simulations are shown in Table 1.

## 3. Materials and Methods

The three-dimensional structure of the target SARS-CoV-2 M^pro^ enzyme was retrieved from the crystal structure deposited in the Protein Data Bank by Jin et al. (PDB accession code 7BQY). The protein structure was prepared by the removal of water molecules and cocrystal ligand, leaving only the amino acid residues. The structures of all the 135 molecules composing the library were built using Avogadro 2.0 [39], and the protonation states were calculated using an in-house code, assuming an acidic condition (pH = 5) to satisfy the enzyme requirements. After this step, histidine residues adopt the following states: HIS^41^, HIP^64^, HIP^80^, HIS^163^, HIP^164^, HIE^172^, and HIE^246^. Prior to the docking step, all ligands were subjected to a systematic conformer search using the SCAN program from the general-purpose molecular modeling software TINKER [40]. Each conformer found was then written to its own file (e.g., AR001.001, AR002.002), and the TINKER’s program ANALYZE was then used to calculate the Total Potential Energy and energy components of each conformer. The lowest energy conformation of each ligand was refined using the semiempirical quantum chemistry program MOPAC7 Baker’s Eigen Following method, using the semiempirical Hamiltonians PM3 (Modified Neglect of Diatomic Overlap, Parametric Method Number 3) [41]. The ligands geometry optimization uses an RMS gradient of 0.0100 as an acceptance criteria. The ligands and the SARS-CoV-2 M^pro^ model were processed using the AutoDock Tool Kit (ADT9 [42] to obtain the PDBQT (Protein Data Bank, Partial Charge (Q), & Atom Type (T)) coordinate files containing the information needed by AutoGrid and AutoDock, namely polar hydrogen atoms, partial charges, correct atom types, and information on the articulation of flexible molecules. In particular, Gasteiger–Marsili charges [43] were loaded in ADT (Auto Dock Tools), and solvation parameters were added to the final structures using the AddSol utility.

Virtual screening experiments were performed using the blind docking mode of the AutoDock Vina software [44]. Individual runs were performed for each ligand conformer, ranking the resulting docked poses using a built-in scoring function. The center of the grid box (5.98, 4.80, 22.29 Å) locates the near center of mass (COM) of the residues of the catalytic dyad (H^41^ and C^145^), and the size of the box (40 × 40 × 40 Å) was determined to sufficiently cover the active site), with a spacing of 0.01 Å. The screening results were analyzed using personal Python scripts, selecting for the next phase only those compounds (poses) capable of meeting a set of criteria.

In practice, the complexes were analyzed to determine the presence of interactions with at least one of the two residues of the catalytic dyad, together with the possibility of coming into contact with at least one residue of each of the four sub-pockets (S1^I^, S1, S2, and S4). These criteria were chosen in the hope of identifying scaffolds that, if properly functionalized, could lead to effective SARS-CoV-2 M^pro^ inhibitors. Compounds meeting these criteria represented 11.6% of the initial library and were characterized by a score lower than −9 kcal/mol.

After this selection, compounds with strong interaction with the SARS-CoV-2 M^pro^ were further validated by using the standard molecular docking protocol. For this purpose, the software Autodock 4.2 [45] was chosen, and the Lamarckian Genetic Algorithm was used in order to combine global search (Genetic Algorithm alone) to local search (Solis and Wets algorithm) [46]. Each docking consisted of an initial population of 250 randomly placed individuals, a maximum number of 25,000,000 energy evaluations, a mutation rate of 0.02, a crossover rate of 0.80, and an elitism value of 1.0. For the local search, the so-called pseudo-Solis and Wets algorithm was applied using a maximum of 250 iterations per local search. Two hundred fifty independent docking runs were carried out for each ligand. The grid maps representing the system in the actual docking process were calculated with AutoGrid. The dimension of the grid was set to sufficiently cover the active site (60 × 60 × 60), with a spacing of 0.01 Å. The docking results were scored using a modified version of the simpler inter-molecular energy function in AutoDock. The results differing by less than 1.0 Å in positional root-mean-square deviation (rmsd) were clustered together and were represented by the result with the most favorable free energy of binding.

In order to study the stability of the systems, the AR005, AR010, AR047, and AR066 complexes obtained from molecular docking were subjected to molecular dynamics (MD) simulations using the GROMACS 2018 package [47,48]. The CHARMM 36 force field [49] was used to simulate the structures.

A water solvated rectangular box was built by using the TIP3P water model [50] with periodic boundary conditions and optimally oriented to minimize the resulting volume. The solutes were centered in the simulation box with a minimum distance to the box edge of 10.0 Å (1.0 nm). After defining the box, all the systems were solvated using the TIP3P water model and neutralized by adding Na^+^ counter-ions by using the ‘gmx genion’ script (Table 2).

Each solvated system was energy-minimized by two steps using the steepest descent method either until the maximum force was smaller than 1000 kJ mol^−1^ nm^−1^ on any atom or until additional steps resulted in a potential energy change of less than 1 kJ mol^−1^ to reduce undesirable atomic contacts. At first, the positioned restraints with a force constant of 1000 kJ mol^−1^ nm^−1^ were applied to all heavy atoms of the SARS-CoV-2 M^pro^ and ligands, allowing water molecules and counter-ions to relax their position. Second, the restrains on proteins and ligands were released, then allowing all atoms in a system to freely move in turn. Afterward, the energy-minimized systems were equilibrated in three phases with the positioned restraints described earlier. The first step was heating up each system from 50 to 300 K over 50 ps under the NVT condition using the Berendsen thermostat [51,52]. The following step was conducted under the NPT condition at 1 bar pressure over 100 ps using the Parrinello–Rahman barostat [53,54]. Once each system was sufficiently equilibrated around the target temperature and pressure, the positioned restraints were then gradually reduced to zero kJ mol^−1^ nm^−1^ with four rounds of 100 ps-NPT simulations. After the equilibrations, 1.0 ns of unrestrained dynamics production was subsequently performed under the NPT condition with snapshots collected every 1 ps. For all the dynamic runs, the covalent bonds in the system were constrained by the linear constraint solver algorithm [55]. A short-range nonbonded interaction cut-off distance of 1.0 nm was used. The long-range electrostatic interactions were obtained by the particle mesh Ewald method [56,57].

Molecular graphics and analyses were performed with BIOVIA DS Visualize 2019 [58] and Univeristy of California San Francisco (UCSF) ChimeraX, developed by the Resource for Biocomputing, Visualization, and Informatics at the University of California, San Francisco, with support from the National Institutes of Health R01-GM129325 and the Office of Cyber Infrastructure and Computational Biology, National Institute of Allergy and Infectious Diseases [59].

## 4. Conclusions

The recent outbreak of he novel coronavirus disease (COVID-19) has evolved into an unprecedented global pandemic, infecting hundreds of thousands of people worldwide. The absence of available antiviral drugs or vaccines to control the infection has resulted in an urgent need for effective drugs to treat the virus.

The main protease (M^pro^) of SARS-Co-V-2 is one of the potential targets due to its essentiality in viral replication. The active site of M^pro^ is highly conserved among all CoVs and is composed of four pockets (S1^I^, S1, S2, and S4), which can easily accommodate a wide selection of fragments. Thus, the ideal candidate should be large enough to occupy the pocket and properly functionalized to improve the interaction with the residues of the other pockets of the protease.

Using a combined approach of virtual screening and molecular docking techniques, we have screened an in-house library mainly composed of natural compounds and structural mimics isolated or synthesized by our research group. This library consisted of structurally diverse small molecules to cover a wide range of biologically relevant chemical spaces. Virtual screening experiments were performed using the blind docking mode of the AutoDock Vina software. Among the tested molecules, a small number of compounds, which showed the best interaction with the protein, were selected to be re-scored by using the standard molecular docking protocol as implemented in AutoDock. The stability of the complexes, thus obtained was confirmed through a 1.0 ns MD study.

The most promising candidates showed strong ability to fit into the protein binding pocket and to reach the catalytic dyad. From the result, the findings emerged that the size and shape of the molecule play an essential role for the interaction with the protein. To be effective, molecules do not necessarily have to be bulky, such as CPT-RGD or the acidic metabolite of *Stachys ehrenbergii*. Molecules such as lamellarins, although more compact, are able to interact with all the pockets of the site, showing a chemical-physical profile typical of drug-like molecules. Likewise, the derivatives of leopolic acid, although interacting weakly with the S2 pocket, show very effective interaction with the catalytic dyad and a favorable chemical-physical profile.

A careful analysis of the geometry and interactions between these key molecules and the enzymatic site could prove essential to obtain better inhibitors. In this regard, CPT, leopolic acid, and lamellarin D derivatives seem to be very promising scaffolds. Since these compounds have been validated by molecular docking and by the analysis of their properties, there is a high probability that at least one of the most promising molecules may be bioactive, and it may be worth further investigation.

## Figures and Tables

**Table 1:** Average values of Root-Mean-Square Deviations (RMSD) and Root-Mean-Square Fluctuations (RMSF) of the four studied complexes.

Cpd	RMSD	RMSF
AR005	0.13 ± 0.02	0.07 ± 0.02
AR010	0.15 ± 0.02	0.08 ± 0.03
AR047	0.12 ± 0.02	0.06 ± 0.03
AR066	0.18 ± 0.02	0.09 ± 0.03

**Table 2:** Characteristics of the simulated systems.

Cpd	Box Volume Å3	TIP3P Molecules	Na + Ions
AR005	390.817	10977	1
AR010	391.206	10997	1
AR047	391.835	10996	1
AR066	391.858	10897	3

## Supplementary Materials

The following are available online, Table S1: PDB structures (as of July 1, 2020) complexed with Ligands of Interest (LOI), as reported in the COVID-19/SARS-CoV-2 Resources page of the Protein Data Bank. Columns refer to the PDB code, release date, target name(s) and LOIs (ID, Molecular Weight, and InCHIKey), Table S2: List of compounds subjected to virtual screening, and Table S3: Chemical-physical characteristics of the compounds present in the library, calculated using the DataWarrior software v5.2.1 [60]. Values in the Drug-like column are 1 if and only if the number of violations of Lipinski’s Rule of Five [61] are < 2, otherwise zero. Likewise, values in the Lead-like column are 1 if and only if the number of violations of Oprea’s lead-like test [62] are < 2, otherwise zero.
